# Aliquat 336 in Solvent Extraction Chemistry of Metallic ReO_4_^−^ Anions

**DOI:** 10.3390/molecules29102257

**Published:** 2024-05-11

**Authors:** Maria Atanassova, Zhanina Petkova, Vanya Kurteva

**Affiliations:** 1 Department of General and Inorganic Chemistry, University of Chemical Technology and Metallurgy, 8 Kliment Okhridski Blvd., 1756 Sofia, Bulgaria; 2 Institute of Organic Chemistry with Centre of Phytochemistry, Bulgarian Academy of Sciences, Acad. G. Bonchev Street, Block 9, 1113 Sofia, Bulgaria; zhanina.petkova@orgchm.bas.bg (Z.P.);

**Keywords:** liquid–liquid extraction, Aliquat 336, ReO_4_^−^, organic diluents, selectivity

## Abstract

A study of the liquid–liquid extraction of ReO_4_^−^ anions from hydrochloric acid solutions using the ionic liquid Aliquat 336 (QCl: trialkyl(C_8_–C_10_)methylammonium chloride) via the well-known method of slope analysis along with the determination of the process parameters is presented. This study employs CCl_4_, CHCl_3_ and C_6_H_12_ as diluents. This study was carried out at room temperature (22 ± 2) °C and an aqueous/organic volumetric ratio of unity. The ligand effect on the complexation properties of ReO_4_^−^ is quantitatively assessed in different organic media. The organic extract in chloroform media is examined through ^1^H, ^13^C and ^15^N NMR analysis as well as the HRMS technique and UV-Vis spectroscopy in order to view the anion exchange and ligand coordination in the organic phase solution. Final conclusions are given highlighting the role of the molecular diluent in complexation processes and selectivity involving ionic liquid ligands and various metal s-, p-, d- and f-cations. ReO_4_^−^ ions have shown one of the best solvent extraction behaviors compared to other ions. For instance, the Aliquat 336 derivative bearing Cl^−^ functions shows strongly enhanced extraction as well as pronounced separation abilities towards ReO_4_^−^.

## 1. Introduction

Rhenium is classified as a critical metal of significant industrial importance due to its valuable physicochemical properties including the high melting point (3 182 °C) and a large number of stable valence states, and consequently, it is an integral component in so-called specialist materials such as high-performance alloys, catalysts, nanomaterials, etc. [1]. In the world, its distribution today like all others is evaluated as critical and so valuable metals present an uneven situation. Currently, thirty-four elements of the periodic table have been designated by the European Union as “critical” raw materials of strategic importance to the European economy and of high supply risk. As a matter of fact, the average concentration of rhenium in the Earth’s crust is just one part per billion (ppb) and is not included this list [2,3]. On the other hand, the maintenance and development of the European economy and the implementation of a smooth transition to renewable energy sources and waste-free technologies are highly dependent on a number of mineral raw materials. Despite partial successes in recycling, the only source unfortunately remaining is mining and extraction from deposits. Therefore, it is necessary to emphasize and draw attention in the scientific research field to the issue of the separation and extraction of rhenium from its associated minerals or related secondary solutions. In general, rhenium is mostly contained in copper sulfide, molybdenum minerals, rare earth ores or niobium–tantalum-containing ores. At present, the method of recovering this metal mainly includes solvent extraction techniques and separation to obtain over 50% of rhenium produced globally [2,3]. As an example, Muruchi et al. have proposed a sustainable and flexible approach for the extraction and separation of rhenium and molybdate species based on a polymeric aqueous two-phase system, in which the high metal content in concentrated copper effluents is directly used as the driver of phase demixing [4]. Moreover, it was found that the resulting process achieves very high separation yields and relies exclusively on cheap chemicals with relatively low toxicity. Thus, it could serve as a more sustainable alternative for the extraction of both metals Re and Mo from copper mine effluents.

In fact, metals and metalloids with high oxidation states exist as oxoanions in water [5]. Thus, the design of functional ligands with a suitable size, shape, electronegativity and hydrophobicity which can reorganize these oxoanions, especially toxic ones, is a significant task to attain effective extraction from the viewpoints of environmental monitoring and protection. Rhenium is known to be a non-radioactive congener of Tc and is applicable as its substitute owing to their similar electronic configuration, stereochemistry and thermodynamic properties as well [6,7]. In essence, studying the technology for separating and extracting rhenium is significant for promoting its industrial applications and the safe disposal of spent fuel [8,9,10]. Therefore, perrhenate anions have adsorption characteristics like those of perchlorate and perthechnate anions (contaminants that cause major environmental concerns), so they can serve as their surrogates. Further, rhenium(VII) is usually present in real technology solutions containing a rather high concentration of sulfuric acid in the form of the perrhenate ion ReO_4_^−^ [11,12]. The equilibria and dynamics of rhenium uptake from innovative materials with anion exchangers have also been studied [13,14]. On the other hand, rhenium(VII) could be recovered from solutions of this kind with anion-exchange extraction agents (aliphatic amines and their salts) or neutral reagents [15,16]. The degree of efficiency of metal ions extracted at the same experimental conditions using amine-based extractants usually follows the order as follows: quaternary > tertiary > secondary > primary. For example, Gerhardt et al. have investigated for the solvent extraction of rhenium various organic extractants, namely tri-*n*-octylamine, bis-isododecylamine, pyridine, Aliquat 336, tributyl phosphate, trioctylphosphine oxide, ethyl xanthate and mesityl oxide [17]. Undoubtedly, among the ionic liquid compounds scientifically investigated the most intensively in the last two decades not only in the field of the liquid–liquid extraction and separation chemistry of metallic species, the one that stands out is Aliquat 336 [18,19]. This colorless viscous liquid compound (0.884 g/cm^3^ density) is a water-insoluble quaternary ammonium salt (Q^+^Cl^−^: Q^+^ is the cation) based on the tricaprylmethylammonium cation [C_25_H_54_N^+^] combined with the chloride [Cl^−^] anion. Nowadays, focusing on the employment of more benign and sustainable technological processes, this fascinating liquid chemical has already found a large-scale application in the field of solvent extraction chemistry: extracting a compound, additive, modifier or organic phase (2 in 1) [20]. For example, the synergistic extraction of lanthanoids with a chelating ligand like thenoyltrifluoroacetone (HTTA) or 1-phenyl-3-methyl-4-benzoylpyrazol-5-one (HP) in combination with Aliquat 336 in the role of a synergistic agent has been studied in detail by Atanassova and Dukov [21,22,23,24,25,26,27]. It has been reported that lanthanoids form anionic complexes of the type Q^+^[Ln(TTA)_4_]^−^ in the organic phase. The role of the quaternary ammonium salt anion is important because anionic complex formation depends on the breaking of the bond between the cation and anion of the liquid salt during the extraction process. Taking into account that the bond energy between the cation and anion increases in the order Cl^−^ < NO_3_^−^ < ClO_4_^−^ [28,29], it is clear why lanthanoid extraction decreases in that order exactly. It has been established that the change in chloride with a perchlorate anion in Aliquat 336 causes a significant decrease in the overall equilibrium constant (3–4 orders). This fact was explained by the authors again with the stronger bond between the cation and the anion in the QClO_4_ molecule which makes the formation of anionic complexes Q^+^[Ln(PMBP)_4_]^−^ more difficult. It was found that the synergistic extraction increases in the sequence CHCl_3_ < C_6_H_6_ < CCl_4_ < C_6_H_12_. As a whole, the increase in the diluent solvating ability hinders the extraction process. Further, the values of the thermodynamic functions have been determined for the extraction of Pr, Gd and Yb with HTTA and Aliquat chloride in CCl_4_, C_6_H_6_ and CHCl_3_ in the interval 288–318 K [25]. The values of ∆*H*, ∆*S* and ∆*G* have been established to be negative for the organic phase synergistic reaction. So, the formation of the anionic complex in the organic phase is favored by the negative enthalpy changes. Perhaps the main disadvantage of this liquid compound, which must be noted, is that it is very viscous; for that reason, it inevitably requires the use of an organic diluent influencing the greenness of the process.

The object of the scientific work undertaken is to present the investigation on the solvent extraction of ReO_4_^−^ ions by the ionic liquid Aliquat 336 diluted in three diluents, CHCl_3_, CCl_4_ and C_6_H_12_, with the goal to elucidate the nature of the extracted complexes into the organic phase as well as to clarify the role of the diluent when its polarity decreases. In addition, the selectivity of Re(VII) and 37 other chemical elements in the periodic table is also studied in detail.

## 2. Results and Discussion

### 2.1. Solvent Extraction Study of ReO_4_^−^ Anions Applying Aliquat 336: Effect of pH, Ligand Concentration and Organic Diluent

**Figure 1:**
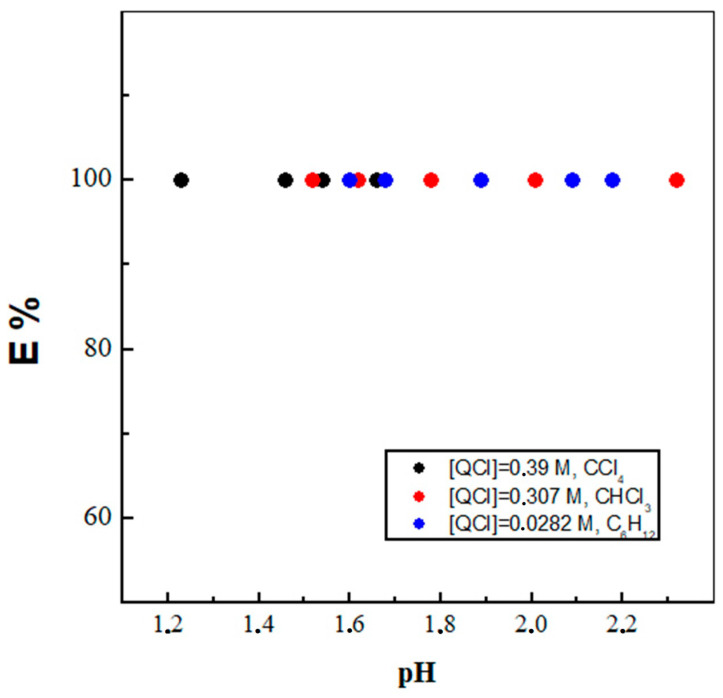
The extraction behavior of ReO_4_^−^ as a function of pH_eq_ in the aqueous phase.

In fact, one ideal representative of ionic liquid compounds is the quaternary ammonium salt, having an industrial nickname of Aliquat 336 (trialkyl(C_8_–C_10_)methylammonium chloride, m.p.: −20 °C; viscosity: 1500 mPa·s). Over and above that, it is an intensively investigated molecule from the broad family of IL compounds in academic fundamental research, with essential application for ion-pair complexation in the mining industry for the role of the extractant [19]. Therefore, it is not a surprise that numerous scientific articles dealing with the evaluation of its coordination ability towards f-elements have also appeared [18,30,31]. However, due to its high viscosity, this IL is mostly used as dissolved in organic molecular diluents, as already mentioned. Additionally, it was found that the solvent extraction mechanism is based on anion exchange and hence strongly depends on the composition of the aqueous phase as well [27,32,33]. The extraction behavior of QCl (Q^+^ is the quaternary ammonium salt cation) towards ReO_4_^−^ as a function of the pH in the aqueous phase using the studied three organic diluents is shown in Figure 1. In all three investigated solvent systems, the extractability of ReO_4_^−^ reaches a maximum value in the pH range from 1.2 to 2.3, i.e., 100%. What is good to pay attention to is that a diluent like CCl_4_ likely prefers a more acidic extraction (aqueous) medium than the other two diluents. Shimojo et al. have recently found that the tripodal ligand *N*,*N*,*N′*,*N′*, *N″*,*N″*-hexa-*n*-octylnitriltriacetamide, composed of three amide groups and a tertiary amine in isooctane medium, quantitatively extracted TcO_4_^−^ and ReO_4_^−^ anions in the pH range from 1.0 to 2.5 [8]. Moreover, it was reported that after five cycles of both forward and back extraction, the ligand retained its high extraction ability for ReO_4_^−^ in the aqueous phase, and the presence of or increase in NO_3_^−^ anions in the aqueous phase unfortunately inhibited the ReO_4_^−^ transfer. The effect of the composition of the organic and aqueous phases on the recovery of rhenium(VII) by triisooctylamine dissolved in 1-octanol or 2-ethyl-1-hexanol was analyzed by Kasilkov and Petrova [34]. Furthermore, the established decrease in *D*_Re_ on passing from weakly acidic to more concentrated solutions is found to be probably due to a change in the state of the ligand salt in the organic phase under conditions of an increasing concentration of H_2_SO_4_ from 0.1 to 9 mol/dm^3^ in the aqueous phase. The observed effect was accounted for by the competing extraction of the mineral acid by the amine, which hinders the rhenium(VII) solvent extraction. The effect of salt (Na^+^) or acid anions was investigated by Zhou and co-researchers that indicated a weaker competition of SO_4_^2−^ or Cl^−^ over ReO_4_^−^ than that of NO_3_^−^ attributed to the largest ΔG_h_ (standard Gibbs hydration energy) value of NO_3_^−^ among the three anions [9]. Thus, the inhibition of *D*_Re_ caused by other competitive anions followed the order as follows: ClO_4_^−^ > NO_3_^−^ > SO_4_^−^ ≈ Cl^−^. Accordingly, this shows that exactly the chosen chloride anion investigated herein is an ideal candidate and the most suitable for an ion exchange with metal anion species like ReO_4_^−^.

**Figure 2:**
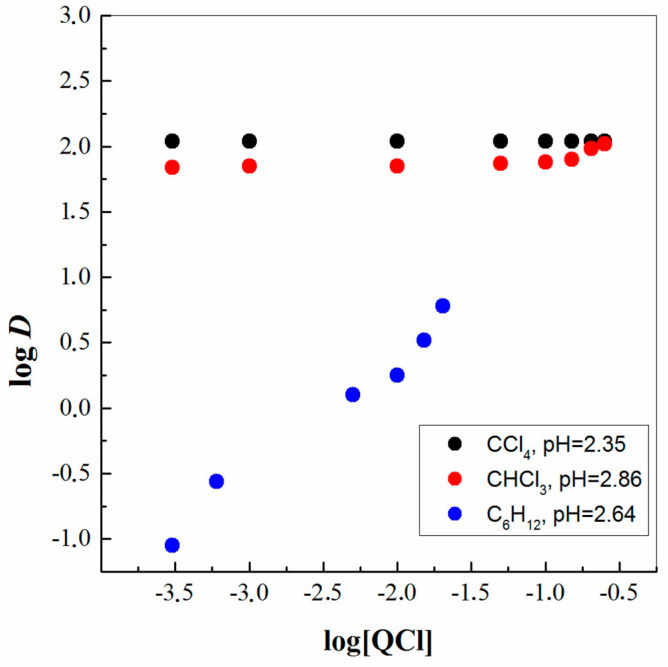
Slope analysis of ReO_4_^−^ extraction by QCl: dependency on extractant concentration.

Further, the mechanism of ReO_4_^−^ solvent extraction with QCl was investigated using the known slope analysis method [27]. In this respect, Figure 2 represents logarithmic plots of the distribution ratio (*D*) of ReO_4_^−^ using QCl as a function of the logarithmic extractant concentrations at a constant pH of the aqueous phase in three different organic diluents. It is evident from the obtained results that this liquid compound is quite a strong extractant for metal anions. Therefore, an indisputable advantage is that very small concentrations of the applied extractant QCl completely extract the metal Re(VII) particles from the aqueous to the organic phase. Thus, it is unnecessary for the ligand to be in excess or at a concentration greater than a 1:1 ratio to the metal. Unfortunately, only in a diluent like C_6_H_12_ is it possible to report and calculate an approximate slope, i.e., ca. 0.88. Of course, the concentration selected for analysis when using this diluent is also smaller. This result implies that one molecule of QCl ([R_3_CH_3_N^+^][Cl^−^]) participated in the ReO_4_^−^ extraction and 1:1 QCl: ReO_4_^−^ complexes are formed in the organic phase with possible stoichiometry Q^+^ReO_4_^−^. Thus, the reaction mechanism in the organic phase is via ion-exchange mode, and the solvent extraction of metallic anions is much more efficient in solvent systems with chlorine-containing diluents. That is, the whole body of the date obtained, combined with previously published evidence, makes it possible to represent the extraction of Re(VII) with a new formation connected with breaking of the bond between the cation ([R_3_CH_3_N^+^]) and the anion ([Cl^−^]) of the IL salt.

Therefore, the solvent extraction process of the metallic anionic species ReO_4_^−^ can be expressed with equilibrium as follows:

[R_3_CH_3_N^+^][Cl^−^]_(o)_ + ReO_4_^−^_(aq)_ ⇌ [R_3_CH_3_N^+^][ ReO_4_^−^]_(o)_ + Cl^−^_(aq)_ (1)

where [R_3_CH_3_N^+^][Cl^−^] denotes the ligand Aliquat 366, and the subscripts “aq” and “o” denote aqueous and organic phases, respectively.

In general, the solvent extraction mechanism involving the Aliquat 336 molecule alone is based on the anion exchange or ion association reaction mode of behaviors with metal chloride or sulfate species and somehow strongly depends on the chemical composition and pH of the aqueous phase (formation of different metal species) [18,35].

**Figure 3:**
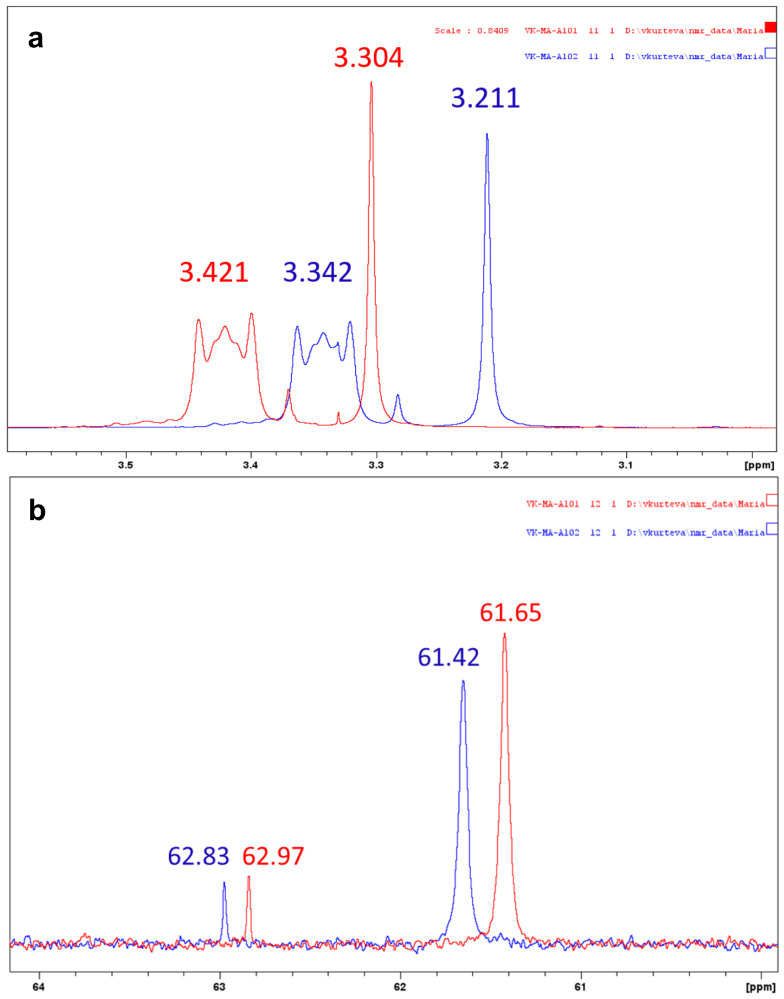
The signals for *N*-methyl and *N*-methylene groups in proton (**a**) and carbon (**b**) spectra of QCl (blue) and QReO_4_ (red) in CDCl_3_.

An additional analysis is undertaken to estimate the degree of anion exchange upon solvent extraction (ligand to metal species concentration ratio 1:1); the latter was performed in deuterated chloroform, and the solutions before and after the extraction of ReO_4_^−^ with the QCl ligand are analyzed by proton, carbon and nitrogen NMR spectra. The signals for methyl and methylene groups neighboring nitrogen are shifted downfield in proton spectra upon anion replacement, 3.211 and 3.342 vs. 3.304 and 3.421, respectively (Figure 3a). The opposite shifting is observed in carbon spectra (Figure 3b), while nitrogen chemical shifts are almost identical (Table 1). Therefore, the fact that these particular chloride signals are not detected in the organic perrhenate spectra indicates that the anion is completely exchanged during the solvent extraction process.

**Figure 4:**
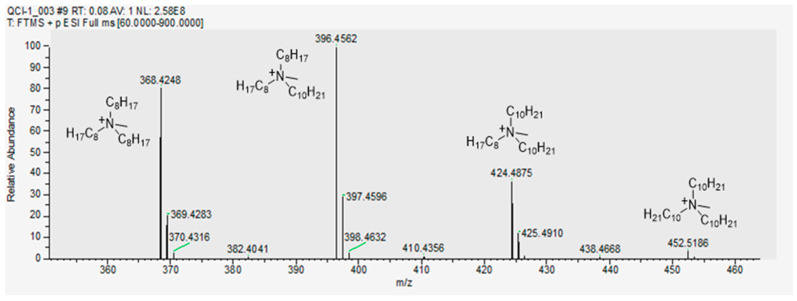
HR–MS (ESI^+^) *m*/*z* calcd. for C_25_H_54_N^+^ [M]^+^ 368.4251, found 368.4248, ∆ = −0.3 mDa; calcd. for C_27_H_58_N^+^ [M]^+^ 396.4564, found 396.4562, ∆ = −0.2 mDa; calcd. for C_29_H_62_N^+^ [M]^+^ 424.4877, found 424.4875, ∆ = −0.2 mDa; calcd. for C_31_H_66_N^+^ [M]^+^ 452.5190, found 452.5186, ∆ = −0.4 mDa.

**Figure 5:**
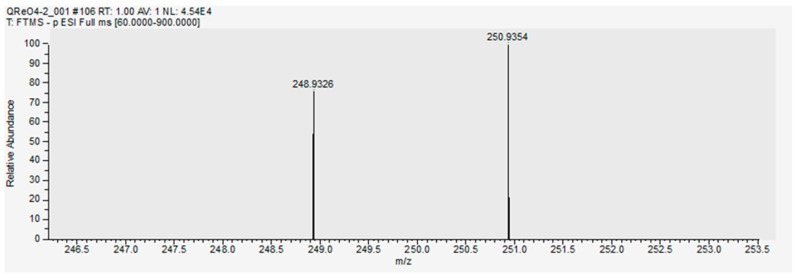
HR–MS (ESI^−^) *m*/*z* calcd. for O_4_Re^−^ [M]^−^ 250.9349, found 250.9354, ∆ = 0.5 mDa.

In fact, Aliquat 336 is typically specified by the supplier to be a trialkylmethylammonium ion consisting of a mixture of C_8_ and C_10_ chains regarding the three alkyl residues with C_8_ predominating. As can be seen from the spectrum in Figure 4, the analysis of the supplied Aliquat 336 by positive ion mode HR-MS disclosed its actual composition. Therefore, four groups of signals with different *m*/*z* ratios and varying intensities could be clearly distinguished and assigned to trioctylmethylammonium (A336^+^_C25_), dioctyldecylmethylammonium (A336^+^_C27_), didecyloctylmethylammonium (A336^+^_C29_) and tridecylmethylammonium (A336^+^_C31_). Note the increasing carbon number in this order from C_25_ to C_31_ as indicated by the subscripts. The observed signal pattern aligns with previously reported ESI-MS data in the literature [36]. The new anion component of the quaternary ammonium cation after the solvent extraction process is shown in Figure 5. It can be seen that the anion ReO_4_^−^ is present approximately in equimolar amounts to the cation Q^+^ (molar ratios close to 1) indicating a quantitative exchange of the chloride counterion of the ligand by the target metallic anion in the course of the solvent extraction process.

**Figure 6:**
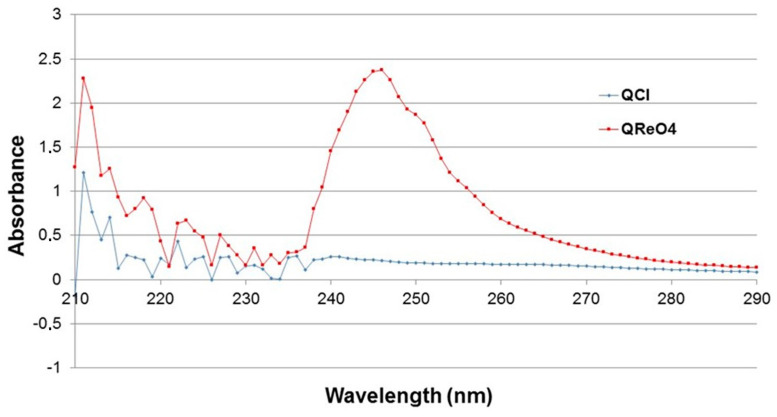
UV–Vis absorption spectra of the formed Q^+^ReO_4_^−^ species extracted by Aliquat 336 from the aqueous solution and the ligand.

In addition, the UV-Vis absorption spectra of the loaded organic phase (CHCl_3_) after the solvent extraction process and the organic liquid phase before extraction were both measured as well. The representative peak for the ReO_4_^−^ anion at wavelength 246 nm [14] was clearly observed (Figure 6), indicating its quantitative transfer from the aqueous phase through an anion exchange mechanism also confirmed by this analysis.

### 2.2. Solvent Extraction and Selectivity across the Chemical Elements in the Periodic Table Applying Aliquat 336

**Figure 7:**
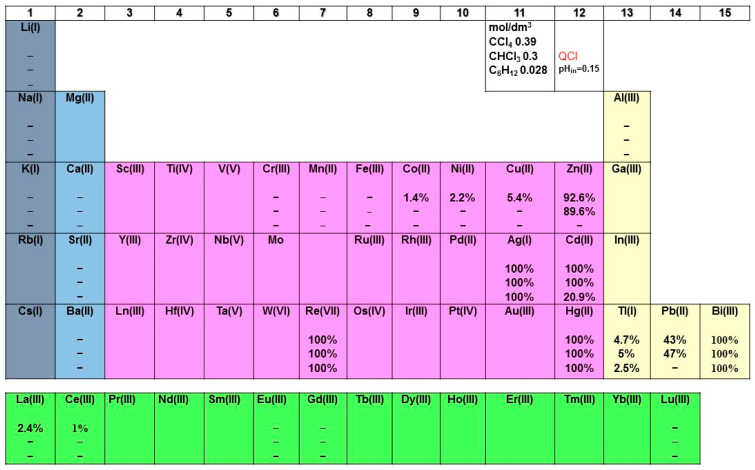
The extraction performance of the Aliquat 336 ligand towards 26 metal ions. The reported extractability values (%) represent the average of three measurements, with deviations of less than 5%.

Generally, as already mentioned, rhenium is present in very low concentrations in the Earth’s crust (0.4 mg·t^−1^) [37]. Additionally, it is produced primarily as a byproduct of molybdenum and copper solvent extraction regardless of the great environmental costs [38]. It is recovered during the pyrometallurgical processing of molybdenum sulfide and copper sulfide ores. This process traditionally involves removing rhenium (VII) oxide, Re_2_O_7_, from the sulfurous gas phase generated during hearth roasting (in molybdenum processing) and smelting (in copper processing) [39,40,41]. The low-grade molybdenite concentrate (20–45%) is also associated with approximately 0.01% Re values, in addition to a number of impurity elements such as Si (7.45%), Fe (1.62%), Ni (0.17%) and Cu (0.35%) [42]. Therefore, it would be interesting to study the behavior of more metals during their simultaneous solvent extraction and to compare the obtained results with the target metal, i.e., rhenium. In general, the extraction efficiency diminishes in competitive extraction tests with respect to the single species extraction, which is probably due to a multi-ion competition, or crowding effect or both of them [43]. From the research study aimed at evaluating the solvent extraction ability of the Aliquat 336 molecule towards various metal cations, it can be seen that it is not a suitable extractant for alkali, alkaline earth metals, Cr, Fe, Al or, of course, lanthanoids [27,44], as shown in Figure 7. The lanthanoid solvent extraction with the quaternary ammonium salt Aliquat 336 in perchlorate form alone is negligible under the experimental conditions applied in the previous study by Atanassova as well [27]. Secondly, regardless of the diluent used, ligand concentration or cation charge, complete quantitative extraction was achieved in all three solvent systems for ions such as Re, Hg, Bi and Ag (Figure 7). Unfortunately, when using Aliquat 336, the increases in metal extraction percentages decreased selectivity regardless of the diluent, since the obtained extraction percentages are very close. This indicates a decrease in the separation factors and, consequently, selectivity between target metals. However, for two of the cations in group 12, viz. Zn^2+^ and Cd^2+^, changing the diluent leads to a reduction in their extraction process by five times or a lack of it. Moreover, one cannot fail to emphasize the commonly observed better capabilities of the diluent CCl_4_ in extraction systems compared to CHCl_3_ [45,46], as seen for metals such as Co^2+^, Ni^2+^, Cu^2+^ and light 4f-ions. Thus, the efficient and selective leaching of specific metals from minerals or wastes is feasible by using the Aliquat 336 compound. In addition, it can be concluded that the proposed solvent extraction system exhibits highly selective extraction for ReO_4_^−^ in the presence of a large number of diverse ions, as shown in Table 2. The corresponding values of separation factors (SFs) for one solvent system are presented only.

In summary, the 26 studied metal ions could be classified approximately in the following four groups according to the degree of their separation with Re(VII):

(1) Very difficult to separate, SF < 5: Zn, Ag, Cd, Hg and Bi;
(2) Difficult to separate, SF < 100: Pb;
(3) Overall selectivity ≈ 10^3^: Li, Al, Fe, Co, Ni, Cu, Ba, La and Tl;
(4) Overall selectivity ≈ 10^4^−10^5^: Na, K, Ca, Cr, Mn, Sr, Ce, Eu, Gd and Lu.

**Figure 8:**
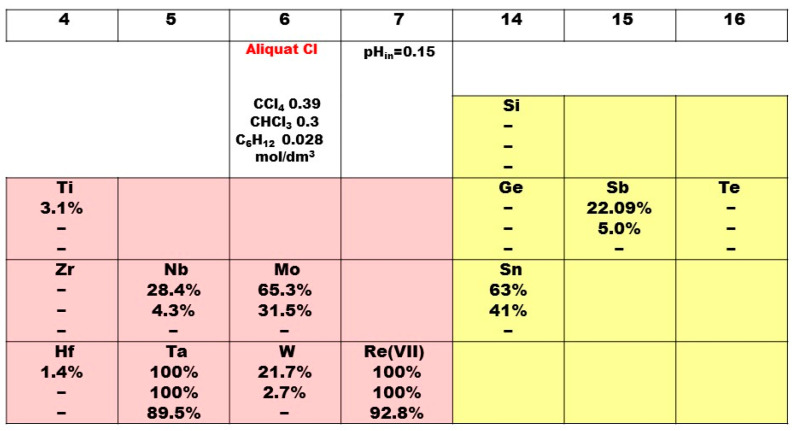
The extraction performance of Aliquat 336 towards 13 metal ions. The reported extractability values (%) represent the average of three measurements, with deviations of less than 5%.

From the additional analysis carried out to investigate the selectivity between rhenium and 12 refractory elements, it is seen that only the C_6_H_12_ diluent and low extractant concentrations are suitable for this scientific purpose separation except only the Re/Ta pair, as shown in Figure 8. Regarding the environmental pressure today in terms of green chemistry, the C_6_H_12_ system may play a role in these efforts so as to create eco-friendly organic solutions, i.e., halogen-free. The solvent system QCl/CCl_4_ is obviously quite effective for the competitive extraction of metal ions, like Re, Ta, Mo and Sn, and their pronounced separation from the rest of the ions in the studied mixture, i.e., Si, Ti, Ge, Sb, Te, Zr, Nb, Hf and W. On that account, the obtained extractability followed the following order: Re ≈ Ta > Mo ≈ Sn > Nb > Sb ≈ W > Ti > Hf > (Zr ≈ Ge ≈ Te ≈ Si). It is worthy to mention as well that when changing the diluent CCl_4_ with polar one CHCl_3_, a sensitive difference is noticed as the extraction ability of the ligand molecule remains at 100% only for Re and Ta ions and sharply decreases for the other ions in the series. Therefore, this know-how could be used in order to obtain a certain selectivity among refractory metals. The diluent in use can influence the solvent extraction efficiency through both chemical and entrainment effects. The use of CCl_4_ as a diluent increases the solvent extraction of Mo and acts more effectively than CHCl_3_ or the influence of aromatic diluent components on the selectivity of the extractant, i.e., C_6_H_12_.

Moreover, in hydrometallurgy, the separation between rhenium and molybdenum ions in aqueous solution is a difficult task owing to their relatively adjacent positions in the periodic table and being in possession of similar chemical properties. One example is the recovery of rhenium and molybdenum from a sulfuric acid leaching solution of molybdenite roasting dust performed by tri-octyl-amine dissolved in kerosene. The extraction efficiency of rhenium and molybdenum was obtained and reported by Kang et al. as 99 and 30%, respectively, using 5 vol% TOA at the solution equilibrium pH of 0 and an O/A ratio of 0.4 [47]. So, the published result clearly shows that a pH equal to 0 is somehow a better experimental condition to separate both metals with the highest separation factor. Further, the extraction of some oxonium anions, TcO_4_^−^, ReO_4_^−^, Cr_2_O_7_^2−^, MoO_4_^2−^ and WO_4_^2−^, from 1 mol/dm^3^ HNO_3_ was performed by a new neutral, tridentate ligand, 2,2′-(methylimino)bis(*N*,*N*-dioctylacetamide) in *n*-dodecane with a reported trend of *D* in the order Tc > Re > W > Mo > Cr [48]. The masking effects of 16 S-, O- and N- donor water-miscible multidentate ligands showing a five- or six-membered ring formation on the solvent extraction of Pd, Ru, Mo and Re from 2 mol/dm^3^ HNO_3_ were studied using the same ligand as well as NTAamide dissolved in *n*-dodecane. However, no masking agent for Re was found in the cited work, contrary to other metals tested [49].

The selectivity of Re(VII) and the twelve refractory ions is studied for the best system, i.e., QCl/CCl_4_, and the results are displayed in Table 3. As a whole, the calculated separation factors obtained for all pairs are extremely high with one exception Re/Ta and are not significantly lower for Mo and Sn combinations. In general, the observed strong selective extractant’s behavior could be discussed as two possible reaction mechanism effects: an anionic, which, in this case, seems to be the determinant, or cationic extraction process followed by a solvation. In other words, this ionic liquid ligand presents a higher selectivity due to its different extraction force towards s-, p-, d- or f-ions. The quaternary ammonium salt, an economically advantageous class of industrial compounds known for decades, will rest a favorable anion-exchange extraction agent in the field of chemical and environmental engineering.

## 3. Materials and Methods

### 3.1. Reagents

All reagents were purchased from Aldrich, Merck and Fluka and were used without further purification. The commercial product Aliquat 336 was obtained from Fluka (Bern, Switzerland). The quaternary ammonium salt was purified before use according to the procedure suggested by Goto [29]. The diluents were CHCl_3_ (Merck, p.a. (Darmstadt, Germany)), C_6_H_12_ (Merck, p.a.) and CCl_4_ (Merck, p.a.). Re standard for ICP 999 mg/dm^3^ ± 4 mg/dm^3^ (Sigma-Aldrich (St. Gallen, Switzerland)) and ICP-MS refractory elements standard—12 components: 10 mg/dm^3^ ± 0.022–0.059 mg/dm^3^ each of Ge, Hf, Mo, Nb, Sb, Si, Sn, Ta, Te, Ti, W and Zr (CPAchem Ltd., Bogomilovo, Bulgaria). All other commercially available analytical-grade reagents were used without any further purification.

A stock solution of rhenium ions (2 × 10^−3^ mol/dm^3^) was prepared from KReO_4_ (Fluka, puriss) by dissolving and diluting with distilled water to the required volume. Hydrochloric acid 37% was used (Merck, p.a.) to adjust the pH of the aqueous solutions added to 0.1 mol/dm^3^ 2-morpholinoethanesulfonic acid (MES) buffer (Alfa Aesar, 98% (Karlsruhe, Germany)).

### 3.2. Solvent Extraction Studies

Extraction experiments were carried out at room temperature by mixing the two immiscible liquid phases in a 1:1 *v*/*v* ratio (1.5 mL) for 1.5 h (1500 rpm), which was sufficient for attaining equilibrium. After the separation of the liquid phases, the concentration of rhenium in the aqueous phase was determined by using ICP-OES spectroscopy (“Prodigy” High-dispersion ICP-OES, Teledyne Leeman Labs, Hudson, New Hampshire, USA). The concentration of the metal ion in the organic phase was obtained by material balance. The reproducibility of distribution ratio (*D*) measurements was generally within 95%. The acidity of the aqueous phase at equilibrium was measured by a pH meter (pH 211 HANNA, Hanna, Smithfield, RI, USA) with an accuracy of 0.01 pH unit. The Re(VII) ion initial concentration was 2.6 × 10^−4^ mol/dm^3^ in all experiments.

For competitive extraction tests, a volume of 2 mL of the prepared aqueous solution containing various M^n+^ metal ions (~3 × 10^−4^ mol/dm^3^ including 3 × 10^−4^ mol/dm^3^ Re(VII)) or various refractory elements ((9 mg/dm^3^) including 2 × 10^−4^ mol/dm^3^ Re(VII)) was equilibrated for 2 h (1500 rpm) with a 2 mL organic phase, which includes the studied ligand molecule, Aliquat 336. After phase separation, the metal ion concentrations in the aqueous solution were determined by ICP-OES.

The distribution ratio (*D*) at equilibrium was calculated as follows:

(2) $$D=\frac{{\left[M^{n+}\right]}_{aq,in}-{\left[M^{n+}\right]}_{aq,f}}{{\left[M^{n+}\right]}_{aq,f}}\times \frac{V_{aq}}{V_{o}}$$

where [*M^n^*^+^]*_aq_*,*_in_* is the concentration of the *M^n^*^+^ ion in the aqueous phase before liquid–liquid extraction tests, and [*M^n^*^+^]*_aq_*,*_f_* is the concentration of the same metal ion in the aqueous phase after extraction. In general, *V_aq_* and *V_o_* are the volumes of the aqueous and organic phase as well as the volumes of two immiscible phases used to perform experiments, herein always a 1:1 *v*/*v* extraction. For instance, duplicate experiments showed that the reproducibility of *D* measurements was generally within 95%.

The extractability (% E) was evaluated as follows:

(3) $$\mathrm{extractability}=\frac{\;{{[M}^{n+}]}_{aq,in}-{{[M}^{n+}]}_{aq,f}}{{{[M}^{n+}]}_{aq,in}}\times 100$$

The metal separation between chemical elements in the periodic table can be estimated using separation factors (SFs) determined as a ratio of the distribution ratios of the two studied metal ions:

SF = *D*_1_/*D*_2_. (4)

### 3.3. NMR, UV-Vis and HRMS Measurements

The NMR spectra of the extract solutions at room temperature are recorded directly after Re(VII) solvent extraction (3 × 10^−2^ mol/dm^3^ Re(VII) and QCl, pH_eq_ = 1.4) on Bruker Avance NEO 400 spectrometers (Rheinstetten, Germany) in CDCl_3_ medium. The chemical shifts are quoted in ppm in δ-values against tetramethylsilane (TMS) as an internal standard. The ^15^N chemical shifts are extracted from two-dimensional ^1^H-^15^N HMBC experiments. The spectra are processed with the Topspin 3.6.3 program.

The mass spectra are recorded (Aliquat 336 and organic phase after Re(VII) solvent extraction (1 × 10^−2^ mol/dm^3^ Re(VII) and QCl in CHCl_3_, pH_eq_ = 1.4) using a Q Exactive Plus Hybrid Quadrupole-Orbitrap Mass Spectrometer from Thermo Scientific (HESI HRMS) in both positive and negative modes. The spectra are processed with the Thermo Scientific FreeStyle program version 1.8 SP1 (Thermo Fisher Scientific Inc., Waltham, MA, USA).

The UV–vis spectra of the extract solution at room temperature is recorded directly after Re(VII) solvent extraction (8 × 10^−4^ mol/dm^3^ Re(VII) and QCl in CHCl_3_ at pH_eq_ = 1.4) using an Analytic Jena Specord 200 Plus spectrophotometer (Jena, Germany) and 10.0 mm path length quartz cuvettes.

## 4. Conclusions

The extraction behavior of Re(VII) by an ionic liquid ligand Aliquat 336 indicates a rapid and spontaneous anion exchange reaction at room temperature. The established stoichiometric ratio in the formed extracted compound in the organic phase is 1:1, with the structure [R_3_CH_3_N^+^][ReO_4_^−^]. The investigation of the effect of the organic diluent on the extracting properties of the ligand revealed that the efficiency of Aliquat 336 dissolved in CCl_4_ was higher than its solutions in CHCl_3_ or C_6_H_12_. Based on the evaluated SF values, it was concluded that Aliquat 336 demonstrated a higher extraction performance and a better separation characteristic particularly with respect to ReO_4_^−^. This study clearly showed the high potential of the investigated affordable ionic liquid compound for the selective separation–recovery of metals such as Re, Ta, Mo, Ag, Hg, Bi, Zn and Cd from multi-component solutions.

## Figures and Tables

**Table 1:** Selected signals in ^1^H and ^13^C spectra and ^15^N chemical shifts (extracted from ^1^H-^15^N HMBC experiment) of QCl and QReO_4_ in CDCl_3_.

Compound	C*H*_3_-N^+^	*C*H_3_-N^+^	C*H*_2_-N^+^	*C*H_2_-N^+^	^15^ *N*
[A336^+^][Cl^−^]	3.211	61.65	3.342	62.97	60.2
[A336^+^][ReO_4_^−^]	3.304	61.42	3.421	62.83	60.4

**Table 2:** Separation factors between Re(VII) and various s-, p-, d- and f-ions extracted with QCl = 0.39 mol/dm^3^ in CCl_4_.

SF	Li	Na	Al	K	Ca	Cr	Mn	Fe	Co	Ni	Cu	Zn	
Re	9 × 10^3^	1 × 10^4^	3 × 10^3^	4 × 10^4^	1 × 10^5^	2 × 10^4^	1 × 10^5^	8 × 10^3^	5 × 10^3^	4 × 10^3^	1 × 10^3^	6.7	
**SF**	**Sr**	**Ag**	**Cd**	**Ba**	**La**	**Ce**	**Eu**	**Gd**	**Lu**	**Hg**	**Tl**	**Pb**	**Bi**
Re	5 × 10^5^	1.09	0.86	5 × 10^3^	3 × 10^3^	1 × 10^4^	7 × 10^5^	2 × 10^4^	8 × 10^5^	2.4	2 × 10^3^	106	4.5

**Table 3:** Separation factors between Re(VII) and 12 refractory ions extracted with QCl = 0.39 mol/dm^3^ in CCl_4_.

SF	Si	Ti	Ge	Sb	Te	Zr	Nb	Mo	Sn	Hf	Ta	W
Re	7.3 × 10^4^	4 × 10^3^	3.3 × 10^4^	464	4.2 × 10^4^	3.8 × 10^4^	332	70	77.4	9.2 × 10^3^	4.6	475

## Data Availability

The original contributions presented in the study are included in the article; further inquiries can be directed to the corresponding author.

## References

[1] Shen Z., Tesfaye F., Li X., Lindberg D., Taskinen P. (2021). Review of rhenium extraction and recycling technologies from primary and secondary resources. Miner. Eng..

[2] Lou Z., Xing S., Xiao X., Shan W., Xiong Y., Fan Y. (2018). Selective adsorption of Re(VII) by chitosan modified with imidazolium-based ionic liquid. Hydrometallurgy.

[3] Qi W., He J., Li M., Zhai M., Zhao L. (2022). Efficient extraction of rhenium through demulsification of imidazolium ionic liquid based microemulsions from aqueous solution. Sep. Purif. Tehcnol..

[4] Maruchi L., Schaeffer N., Passos H., Mendonça C., Coutinho J., Jimenez Y. (2019). Sustainable extraction and separation of rhenium and molybdenum from model copper mining effluents using a polymeric aqueous two-phase system. ACS Sustain. Chem. Eng..

[5] Ogata T., Takeshita K., Tsuda K., Mori A. (2009). Solvent extraction of perrhenate ions with podand-type nitrogen donor ligands. Sep. Purif. Technol..

[6] Xiong C., Yao C., Wu X. (2008). Adsorption of rhenium(VII) on 4-amino-1,2,4-triazole resin. Hydrometallurgy.

[7] Chen X.-R., Zhang C.-R., Jiang W., Liu X., Luo Q.-X., Zhang L., Liang R.-P., Qin J.-D. (2023). 3D Viologen-based covalent organic framework for selective and efficient adsorption of ReO_4_^−^/TcO_4_^−^. Sep. Purif. Technol..

[8] Shimojo K., Suzuki H., Yokoyama K., Yaita T., Ikeda-Ohno A. (2020). Solvent extraction of technetium (VII) and rhenium (VII) using a hexaoctylnitrilotriacetamide extractant. Anal. Sci..

[9] Zhou X., Wu R., Kang J., Fan Y., Huang C., Jin Y., Xia C. (2022). Study on extraction behavior of Re(VII) with bis-triamide extractants. Solvent Extr. Ion Exch..

[10] Pan X., Zu J., Han G., Lin S., Diao J., Xue Y. (2023). Efficient and rapid elimination of ^99^TcO_4_^−^/ReO_4_^−^ from medium-low level acid-wastewater using anion exchange adsorbent. Sep. Purif. Technol..

[11] Xiong Y., Song Y., Zhang P., Wang Y., Lou Z., Zhang F., Shan W. (2017). Adsorption-controlled preparation of anionic imprinted amino-functionalization chitosan for recognizing rhenium(VII). Sep. Purif. Technol..

[12] Hori H., Otsu T., Yasukawa T., Morita R., Ishii S., Asai T. (2019). Recovery of rhenium from aqueous mixed metal solutions by selective precipitation: A photochemical approach. Hydrometallurgy.

[13] Laatikainen M., Virolainen S., Paatero E., Sainio T. (2015). Recovery of ReO_4_^−^ by weakly basic anion exchangers: Modeling of sorption equilibrium and rate. Sep. Purif. Technol..

[14] Atanassova M. (2021). Ordered mesoporous silicas containing imidazolium substructures for solid-liquid extraction of metallic anions ReO_4_^−^. Russ. J. Inorg. Chem..

[15] Travkin V., Antonov A., Kubasov V., Ishchenko A., Glubokov Y. (2006). Extraction of rhenium(VII) and molybdenum(VI) with hexabutyltriamide of phosphoric acid from acid media. Russ. J. Appl. Chem..

[16] Batueva T., Radushev A., Tuktareva T., Degtev M., Nasrtdinova T., Karmanov V. (2010). Rhenium(VII) extraction with 2-ethylhexanoic acid N,N-dialkylhydrazides from acidic solutions. Russ. J. Inorg. Chem..

[17] Gerhardt N., Palant A., Petrova V., Tagirov R. (2001). Solvent extraction of molybdenum (VI), tungsten (VI) and rhenium(VII) by diisododecylamine from leach liquors. Hydrometallurgy.

[18] Dukov I., Atanassova M., Morrison D.A. (2010). High molecular weight amines and quaternary ammonium salts as synergistic agents in the solvent extraction of metal ions with chelating extractants. Handbook of Inorganic Chemistry Research.

[19] Mikkola J.-P., Virtanen P., Sjöholm R. (2006). Aliquat 336—A versatile and affordable cation source for an entirely new family of hydrophobic ionic liquid. Green Chem..

[20] Atanassova M. (2021). Solvent extraction chemistry in ionic liquids: An overview of f-ions. J. Mol. Liq..

[21] Genov L., Dukov I., Kassabov G. (1977). Extraction von europium durch gemische von thenoyltrifluoroaceton and Aliquat 336. Acta Chim. Acad. Sci. Hung..

[22] Dukov I., Genov L. (1980). On the mechanism of the synergistic extraction of lanthanides with thenoyltrifluoroacetone and Aliquat-336S. Acta Chim. Acad. Sci. Hung..

[23] Atanassova M., Jordanov V., Dukov I. (2002). Effect of the quaternary ammonium salt Aliquat 336 on the solvent extraction of lanthanoid (III) ions with thenoyltrifluoroacetone. Hydrometallurgy.

[24] Dukov I., Atanassova M. (2003). Effect of the diluents on the synergistic solvent extraction of some lanthanides with thenoyltrifluoroacetone and quaternary ammonium salt. Hydrometallurgy.

[25] Dukov I., Genov L. (1987). Temperature effect on the synergistic solvent extaction of lanthanoides. Solvent Extr. Ion Exch..

[26] Jordanov V., Atanassova M., Dukov I. (2002). Solvent extraction of lanthanides with 1-phenyl-3-methyl-4-benzoyl-5-pyrazolone. Sep. Sci. Technol..

[27] Atanassova M. (2009). Synergistic solvent extraction and separation of lanthanide(III) ions with 4-benzoyl-3-phenyl-5-isoxazolone and the quaternary ammonium salt. Solvent Extr. Ion Exch..

[28] Ivanov N., Gindin L., Tchichagova G. (1967). Izvestiya Sibirskogo otdeleniya Akademii nauk SSSR. Seriya Khimicheskikh Nauk.

[29] Goto T. (1969). Extraction of lanthanoids by quaternary ammonium salts. J. Inorg. Nucl. Chem..

[30] Knight A., Chiarizia R., Soderholm L. (2017). Extraction selectivity of a quaternary alkylammonium salt for trivalent actinides over trivalent lanthanides: Does extractant aggregation play a role?. Solvent Extr. Ion Exch..

[31] Hoogerstraete T.V., Souza E.R., Onghena B., Banerjee D., Binnemans K. (2018). Mechanism for solvent extraction of lanthanides from chloride media by basic extractants. J. Solut. Chem..

[32] Paatero J. (1974). Studies on the System Aliquat 336 in Xylene-Nickel(II) in Aqueous Chloride Solutions.

[33] Lerum H., Andersen N., Eriksen D., Hansen E., Petersen D., Wibetoe G., Omtvedt J. (2018). Study of cadmium extraction with Aliquat 336 from highly saline solutions. J. Solut. Chem..

[34] Kasilkov A., Petrova A. (2006). Recovery of rhenium(VII) with triisoactylamine from sulfuric acid solutions. Russ. J. Applied Chem..

[35] Stojanovic A., Morgenbesser C., Kogelnig D., Krochler K., Keppler B., Kokorin A. (2011). Quaternary ammonium amd phosphonium ionic liquids in chemical and environmental engineering. Ionic Liquids: Theory, Properties, New Approaches.

[36] Stojanovic A., Lämmerhofer M., Kogelnig D., Schiesel S., Sturm M., Galanski M., Krachler R., Keppler B., Lindner W. (2008). Analysis of quaternary ammonium and phosphonium ionic liquids by reversed-phase high-performance liquid chromatography with charged aerosol detection and unified calibration. J. Chromatogr. A.

[37] Casas J.M., Sepúlveda E., Bravo L., Cifuentes L. (2012). Crystallization of sodium perrhenate from NaReO_4_−H_2_O−C_2_H_5_OH solutions at 298 K. Hydrometallurgy.

[38] Vosough M., Shahtahmasebi N., Behdani M. (2016). Recovery Rhenium from roasted dust through super Para-magnetic Nanoparticles. Int. J. Refract. Hard Met. Hard Mater..

[39] Nebeker N., Hiskey J.B. (2012). Recovery of rhenium from copper leach solution by ion exchange. Hydrometallurgy.

[40] Lessard J., Gribbin D., Shekhter L. (2014). Recovery of rhenium from molybdenum and copper concentrates during the looping sulfide oxidation process. Int. J. Refract. Hard Met. Hard Mater..

[41] Amer A. (2008). The hydrometallurgical extraction of rhenium from copper industrial wastes. JOM.

[42] Jumeja J., Singa S., Bose D. (1996). Investigations on the extraction of molybdenum and rhenium values from low grade molybdenite concentrate. Hydrometallurgy.

[43] Safarbali R., Reza Yaftian M., Zamani A. (2016). Solvent extraction-separation of La(III), Eu(III) and Er(III) ions from aqueous chloride medium using carbamoyl-carboxylic acid extractants. J. Rare Earths.

[44] Li Z., Li X., Raiguel S., Binnemans K. (2018). Separation of transition metals from rare earths by non-aqueous solvent extraction from ethylene glycol solutions using Aliquat 336. Sep. Purif. Technol..

[45] Atanassova M., Kukeva R., Kurteva V. (2023). New sustainable solvent extraction pathways for rare earth metals via oximes molecules. Molecules.

[46] Ramachandra Reddy B., Kumar J., Varade Reddy A. (2006). 3-Phenyl-4-acyl-isoxazolones as reagents for liquid-liquid extraction of tetravalent zirconium and hafnium from acidic chloride solutions. J. Braz. Chem. Soc..

[47] Kang J., Kim Y.-U., Joo S.-H., Yoon H.-S., Kumar J., Park K.-H., Parhi P., Shin S. (2013). Behaviour of extraction, stripping and separation possibilities of rhenium and molybdenum from molybdenite roasting dust leaching solution using amine based extractant tri-octyl-amine. Mater. Trans..

[48] Sasaki Y., Kitatsuji Y., Kimura T. (2007). Highly selective extraction of TcO_4_^−^, ReO_4_^−^ and MoO_4_^−^ by the new ligand 2,2’-(methylimino)bis(N,N-dioctylacetamide). Chem. Lett..

[49] Sasaki Y., Morita K., Shimazaki S., Tsubata Y., Ozawa M. (2016). Masking effects for Mo, Re, Pd and Ru by S and N- donor reagents through MIDOA and NTaamide extraction. Solvent Extr. Res. Dev. Jpn..

